# Making Data Reports Useful During the COVID-19 Pandemic

**DOI:** 10.7759/cureus.9945

**Published:** 2020-08-22

**Authors:** Alfred C Ma, David Ninan

**Affiliations:** 1 Medicine, Mansfield International College, Fullerton, USA; 2 Anesthesiology, Riverside University Health System Medical Center, Moreno Valley, USA

**Keywords:** covid 19, emergency service, data and analytics

## Abstract

During the coronavirus disease 2019 (COVID-19) pandemic, anxiety regarding hospitals resulted in patients risking their lives and not seeking emergency medical care when needed. Early into the pandemic, hospital emergency room utilization plummeted more than 40% in some hospitals, according to the Centers for Disease Control and Prevention. As COVID-19 outbreaks intensified in the Western regions of the country, emergency room census began to increase significantly in the middle of June. Local safety net health care resources were struggling with the increase in emergency room utilization and scrambled to increase patient care capacity, especially their emergency rooms and intensive care units. The data collected during this time is of great value. Unfortunately, it is often poorly reported, overlooked, and ignored when it should be used to make better decisions and allocations. During the pandemic, underserved populations were especially impacted, overwhelming safety net health organizations. The findings from a simple data analysis provide a template for resource acuity among communities and depict the importance of health equity.

## Introduction

There are alarming reports that patients are avoiding medical care during the coronavirus disease 2019 (COVID-19) pandemic even when they need emergency care [1]. Serious sequela, especially neurological complications that are sometimes fatal, has been related to the COVID-19 pandemic in a recent report [2]. Unfortunately, patients avoid hospital emergency room care settings and seek care at less crowded urgent care facilities, often resulting in delay. This is counterproductive in mitigating the chance of exposure to the virus.

When armed with transparent and easy to understand information, health care providers are likely to make informed and fitting decisions. During the COVID-19 pandemic, these decisions may include the proactive allocation of resources, the timely completion of patient discharge processes, and attending to necessary consults and admissions promptly to better allocate already limited resources.

Turning data into intelligence can be critical in making such decisions. Health care organizations that put data-based insight at the center of their strategies optimize their performance and produce better outcomes. This article sets out to promote the analysis of data presented daily to medical staff through the observation of emergency room capacity alert reports over the last several months. The aim is to discuss the variable data analytics that can be related to hospital operational management and preparedness during the pandemic.

## Materials and methods

The hospital studied is a state safety net health organization. It is a 440-bed acute care teaching hospital in the Western region of the United States. It has a 45-bed capacity emergency room and is licensed for 48 intensive care beds.

The number of emergency room visits fell nationwide in March and April after the declaration of a national emergency due to COVID-19 [3]. This study entailed a review of daily capacity alert reports data, as shown in Table 1, which is readily available through email twice a day to providers and hospital staff members. Contained in the report is daily numeric data that included emergency room census and emergency room holding census. All data available from March 2020 to July 2020 was quantitatively analyzed for this study.

**Table 1:** Emergency room capacity alert reporting

Today’s date	July 25^th^, 2020 Saturday 07:00 AM
Emergency room (ER) census	53
Emergency room (ER) hold with bed	1
Emergency room (ER) hold without bed	26
Emergency room (ER) COVID-19 hold without bed	1

Data analysis involved grouping and organization of necessary attributes. In this study, data were accumulated and analyzed using pivot tables and charts in Excel®. Some parameters were calculated to gain further insight. For example, hospital bed availability was estimated by calculation in the following formula:

Estimated hospital bed availability = ER hold with bed - ER hold without bed

The output of the above calculation, when a positive number, can indicate the degree of hospital bed availability. Negative values indicate the number of hospital beds needed to ease emergency room overload.

## Results

**Figure 1:**
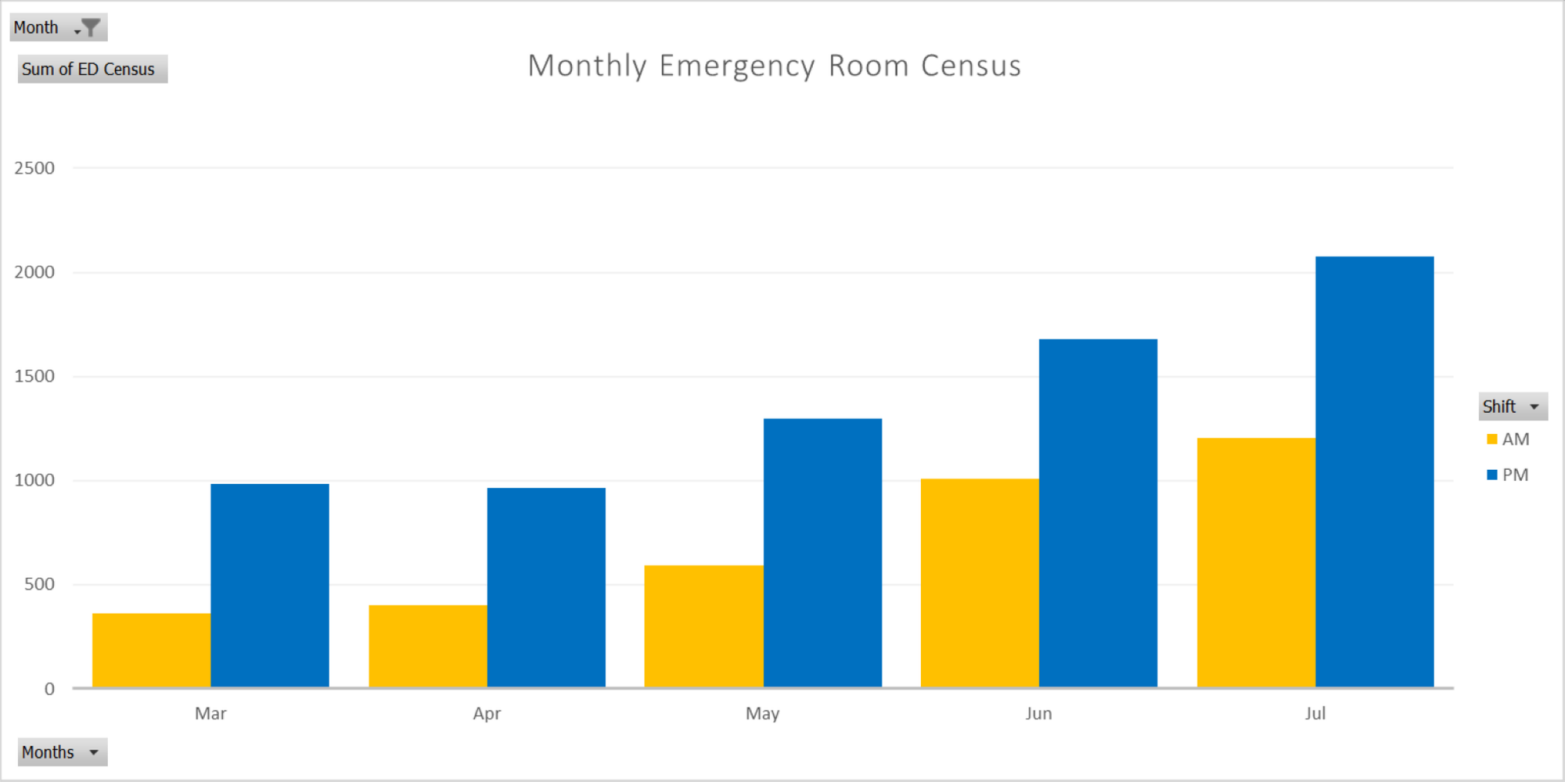
Hospital emergency room average census during months in 2020

In general, emergency room utilization is constantly lower in the morning compared to that in the evening [4]. Before the COVID-19 pandemic, the emergency room census at the hospital was 46% of emergency department capacity in the morning and 102% in the evening. After the declaration of a national emergency for COVID-19 in the US, emergency room utilization during March was 46% of pre-pandemic census in the morning and 75% of pre-pandemic census in the evening. In April, the emergency room census slightly increased to 57% and 79%, respectively. The emergency room censuses returned almost to pre-pandemic levels in May, began to increase significantly in the middle of June, and increased at an accelerated rate into July (Figure 1).

**Figure 2:**
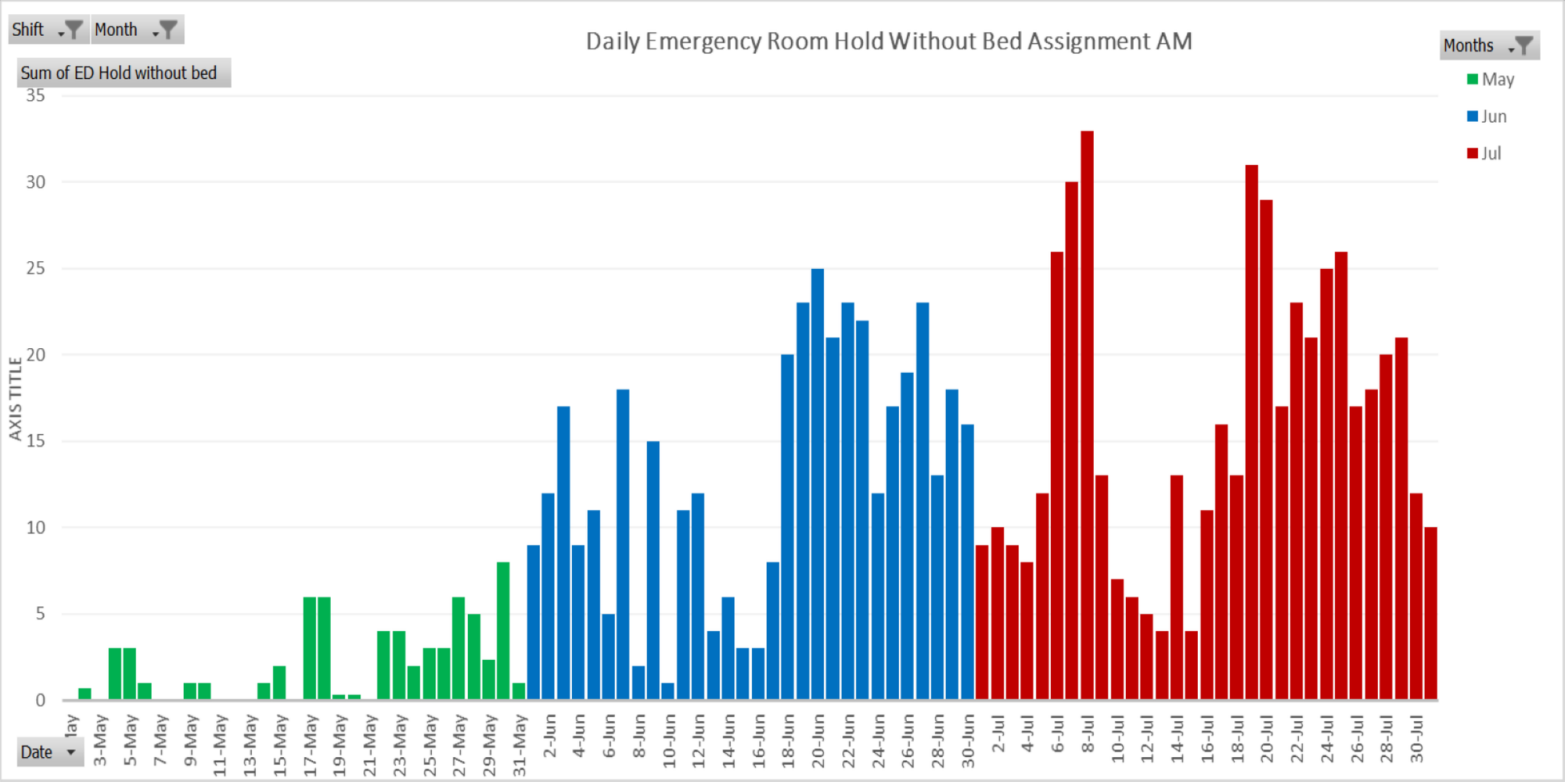
Daily emergency room hold without hospital bed available in the morning

**Figure 3:**
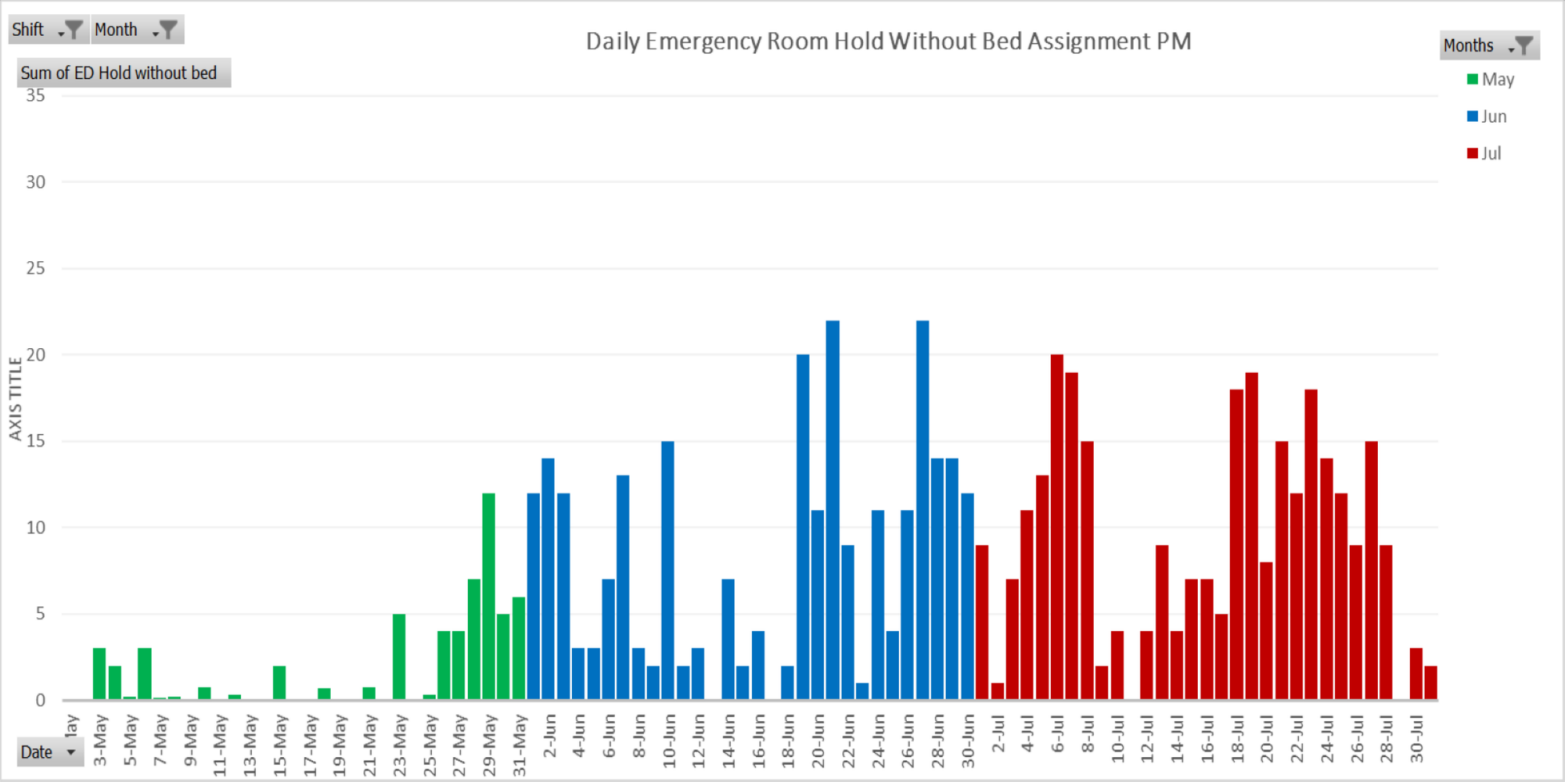
Daily emergency room hold without hospital bed available in the evening

The decrease in emergency room utilization during the months of March and April correlated well with reports nationwide [3]. However, the return of emergency room utilization to pre-COVID levels in May at this safety net hospital seemed early compared to the national average. Higher demand for emergency care in both June and July can be explained not just by months of limited access to healthcare but also by a diversion of public attention away from COVID-19. As seen in Figure 2 and Figure 3, emergency room holds without bed assignment increased in May and continued to amplify in the months to follow. The number of patients retained in the emergency room without bed assignments was significantly higher in the morning (Figure 2) than in the evening (Figure 3). The results depict that admission needs were significantly higher in the morning as compared to the evening. This phenomenon was due to the fact that the allocation of available resources, such as patient discharge and reassignment, was usually far more efficient during the day than at night.

**Figure 4:**
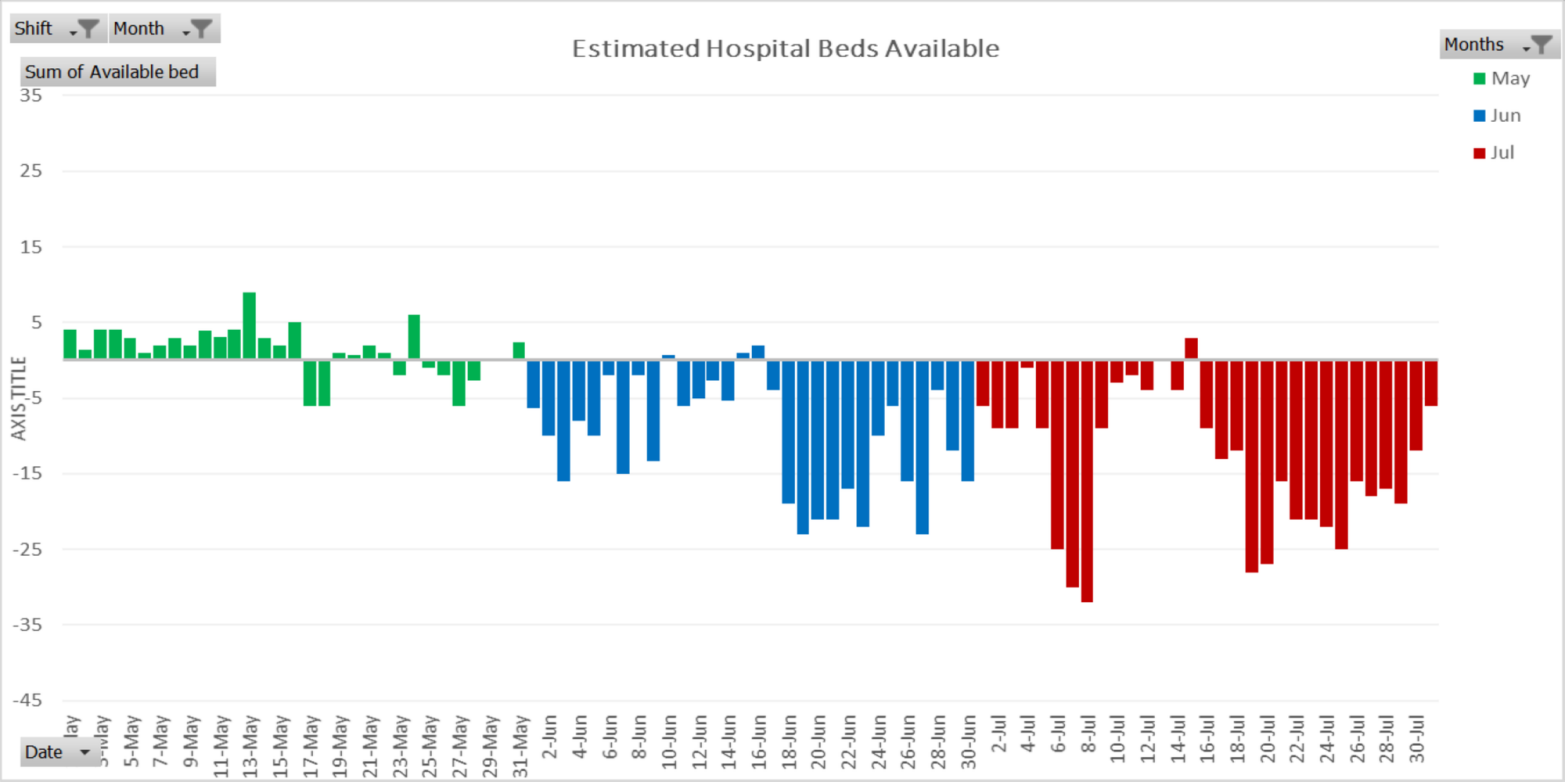
Estimated hospital bed availability

Using the formula explained in the methods section, the estimated hospital bed availability is presented in Figure 4. The scarcity of bed resources began to appear in late May of 2020 and worsened thereafter. The hospital was significantly struggling with bed resources in both June and July.

**Figure 5:**
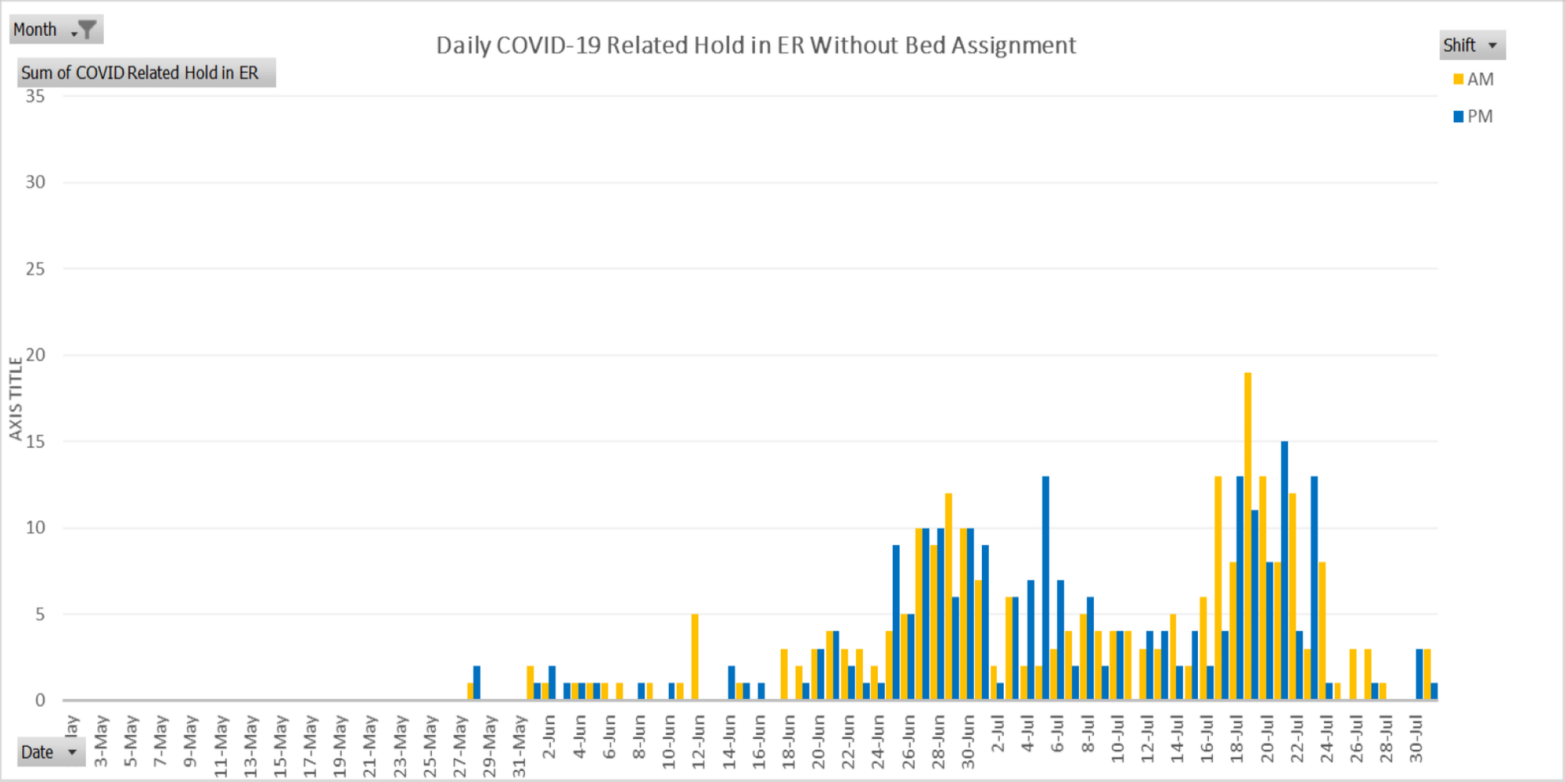
COVID-19 related hold in emergency room without hospital bed available

At the beginning of the COVID-19 pandemic, the startling lack of patients presenting to the emergency room was a prediction that patients could be coming in later and sicker. This was confirmed by the overwhelming increase in the emergency room census in June and July, as shown in Figure 1. Emergency holds without bed assignment can sometimes exceed a shift or even multiple shifts. Thus, quantitatively, the counts of emergency room holds without bed assignment, especially those of COVID-19 cases, were not accurately reflecting the count of actual cases. Yet, the daily analysis of data with visualization tiles easily offered continuous images on the severity of challenges to come (Figure 5).

This information is critical for clinical practitioners to work with administrators to strategize how to counter the many challenges faced during this difficult time. Compared to a daily report that contains a table with merely numeric values, the easy-to-read visual report provides continuous insight into day-to-day census variations in the emergency room. Such reports can be helpful for stakeholders working together to allocate the necessary resources and increase alertness.

## Discussion

These data are valuable for health organizations to gain insight and prepare. Yet, too often, they are ignored. Organizations often express data analytics on a visual dashboard, presuming it is easy to understand. Unfortunately, the dashboard is often overloaded with information and difficult to comprehend. Data expression ought to be specific, complete, and easy to understand. Proper information expressed through dashboard visualization is critical in any application or platform.

Unlike in the business and financial sectors, health data are underutilized [5]. As the nation continues to struggle to eradicate COVID-19, information transparency within health organizations can be critical and educational. The analytics of emergency room capacity alert reports as such, or in any other form, can be helpful for stakeholders to gain better insight into hospitals’ perpetual operational challenges, and, together, they can engage to confront emergencies at various levels.

## Conclusions

The value of daily emergency room capacity alert reports that merely provide numerical values without analysis is extremely limited. For example, patient stasis in the emergency room without bed assignments in the morning was significantly higher than in the evening. This is not merely due to the obvious, such as the availability of beds. It also reflects a scarcity of resources and a lack of preparedness. Visualization of continuous data provides stakeholders with information to be agile in resource allocation and performance. This finding reflects the importance of health equity for the most vulnerable who not only experienced disproportionately negative effects during the crisis but also have very limited access to health care that is delivered mostly by an extremely limited number of safety net health organizations.
